# Supplementary material for: The importance of DNAPKcs for blunt DNA end joining is magnified when XLF is weakened
Source: Nat Commun. 2022 Jun 27;13:3662. doi: 10.1038/s41467-022-31365-6 (PMC9237100; doi:10.1038/s41467-022-31365-6)
Supplement: Supplementary file 1 — Supplementary Information [file 41467_2022_31365_MOESM1_ESM.pdf]

SUPPLEMENTARY INFORMATION

The importance of DNAPKcs for blunt DNA end joining is magnified when XLF is weakened

Metztli Cisneros-Aguirre, Felicia Wednesday Lopezcolorado, Linda Jillianne Tsai,

Ragini Bhargava, and Jeremy M. Stark

Supplementary Table 1. Oligonucleotide list.

Name	Purpose	Sequence (5' → 3', all sgRNA sequences have the initial G nucleotide, regardless of whether it is part of the targeted sequence)
7a	sgRNA	GACCACCCTGACCTACGGCTA
7b	sgRNA	GGCTGAAGCACTGCACGAAT
7a+1	sgRNA	GTAGATATCTTCCTTAGCCGT
GAPDH	sgRNA	GTATAGAAACCGGGGGCGCGG
CD4	sgRNA	GGCGTATCTGTGTGAGGACT
DSB-H	sgRNA	GACGCCCCAGCACTCGTCCGA
DSB-G	sgRNA	GAGCACTGCACGCCGTAGGTC
DSB-L	sgRNA	GCTCTTCGCTATTACGCCAGC
mXlfsg1	sgRNA	GCAAAACAGCAGATCCAAGCA
mXlfsg2	sgRNA	GTCAAGCAGCACCTCCCCTCG
PRKDCsg1	sgRNA	GACTAAAGGCAATTCGTCCTC
PRKDCsg2	sgRNA	GAGACACGTAGTTGTCCAGA
XLFsg1	sgRNA	GTTGGTTTCAGATCTTCAAC
LMNA	sgRNA	GCCATGGAGACCCCGTCCCAG
shXLF	shRNA	CCGGTACCATGGACTTTAGGTATATCTCGAGATATACCTAAAGTCCATGGTA TTTTTG
shCtrl	shRNA	CCGGCCTAAGGTTAAGTCGCCCTCGCTCGAGCGAGGGCGACTTAACCTTAGG TTTTTG
ILL-GAPDH	primer	ACACTCTTTCCCTACACGACGCTCTTCCGATCTACGTAGCTCAGGCCTCAAGA
ILL-CD4	primer	GACTGGAGTTCAGACGTGTGCTCTTCCGATCTACAGTTCCAGTGGGAAATCG
TIDE analysis primers for GFPd2-SV		
LACZ4pcrUP1	primer	AACTTCAAGCTTGGCACTGG
LACZ4pcrDN1	primer	GACAGTATCGGCCTCAGGAA
HYG2pcrUP1	primer	GACGGCAATTTCGATGATG
HYG2pcrDN1	primer	CGAAGCCCAACCTTTCATAG
GFP1pcrUP1	primer	ACGTAAACGGCCACAAGTTC
GFP1pcrDN1	primer	AAGTCGTGCTGCTTCATGTG
qPCR analysis primers for GFPd2-SV		
A site UP	primer	CGTACTACGAGATTTCGATTCCA
A site DN	primer	TTATAAGCTGCAATAAACAAGTTGG
B site UP	primer	TGTCTTGTGCCCAGGAGAG
B site DN	primer	CAACAGATGGCTGGCAACTA
C site UP	primer	TCCCTTTAGGGTTCCGATTT
C site DN	primer	GTTTTCCCAGTCACGACGTT
Actin UP	primer	ACTGGGACGACATGGAGAAG
Actin DN	primer	AGGAAGGAAGGCTGGAAGAG

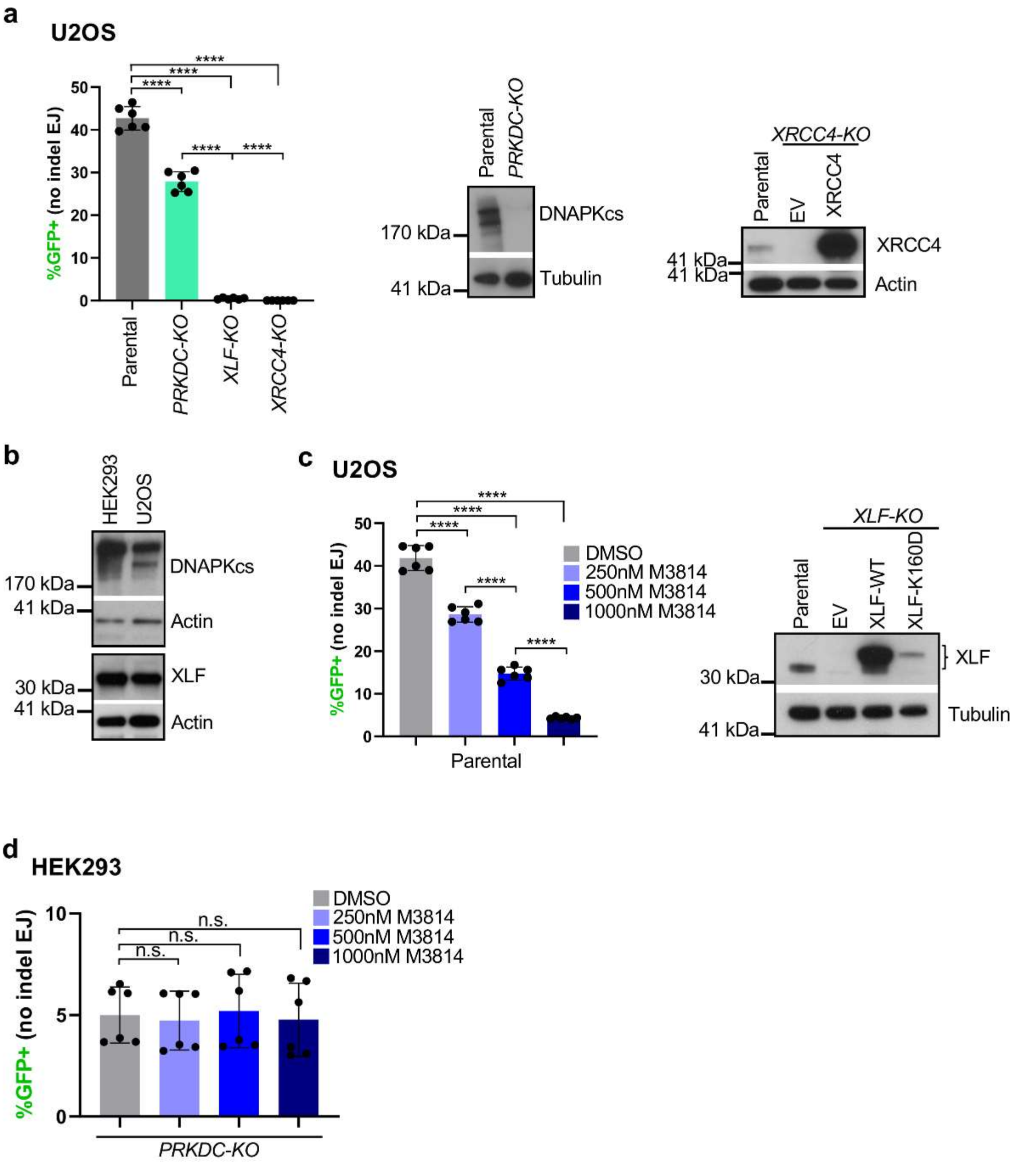

Supplementary Figure 1. DNAPKcs analysis in U2OS cells, comparison of DNAPKcs and XLF levels in U2OS vs. HEK293, and effects of M3814 treatment in *PRKDC-KO* HEK293 cells. (a) DNAPKcs is less important for No Indel EJ vs. XLF and XRCC4 in U2OS cells. n = 6 biologically independent transfections. Statistics with unpaired two-tailed *t*-test using Holm-Sidak correction. ****$P<0.0001$. Immunoblots show levels of DNAPKcs and XRCC4. **(b)** Immunoblots show levels of DNAPKcs and XLF in U2OS and HEK293 cells. Shown are representative immunoblot signals from an experiment performed with two technical replicates. **(c)** M3814 causes a dose dependent decrease for No Indel EJ in U2OS cells. n = 6 biologically independent transfections. Statistics as in (a). ****$P<0.0001$. Immunoblot shows levels of XLF-WT and XLF-K160D in U2OS cells. **(d)** M3814 does not affect No Indel EJ in *PRKDC-KO* cells. n = 6 biologically independent transfections. Statistics as in (a). n.s. = not significant. Data are represented as mean values +/- SD.

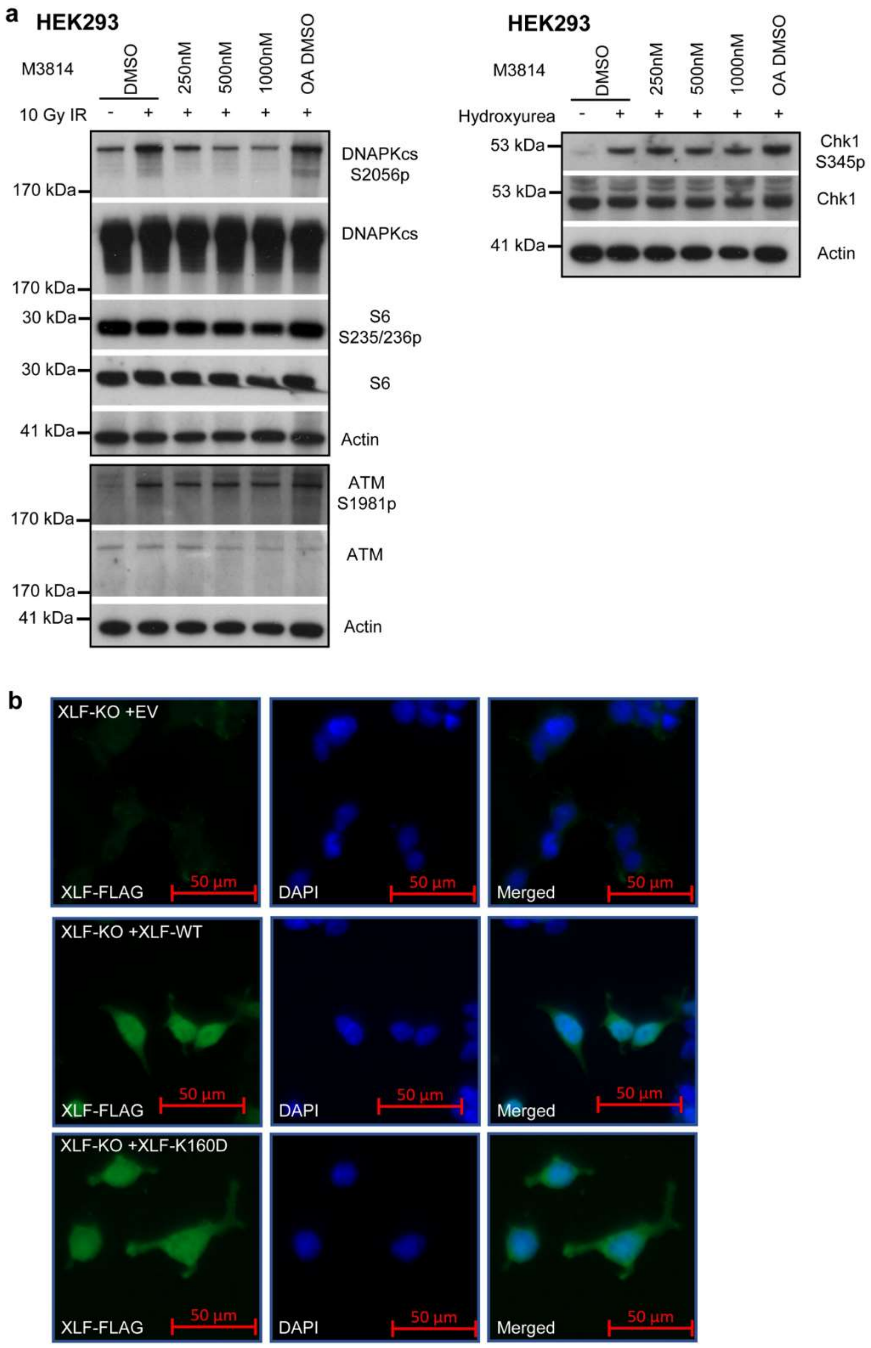

Supplementary Figure 2. Effects of M3814 on a set of phosphorylation events, and evaluation of XLF-WT and XLF-K160D localization. (a) Effects of M3814 treatment on various phosphorylation events using cells treated with M3814, IR, hydroxyurea (5mM), and/or the phosphatase inhibitor okadaic acid (OA, 1 µM). Treatment conditions are the same as in Figure 2b. Shown are immunoblot signals from an experiment performed once for DNAPKcs-S2056p, DNAPKcs, S6-S235/236p (Cell Signaling 2211S, 1:1000), S6 (Cell Signaling 2217S, clone 5G10, 1:1000), ATM-S1981p (Abcam ab81292, clone

EP1890Y, 1:1000), ATM (Santa Cruz sc23921, clone 2C1, 1:1000), Chk1-S345p (Cell Signaling 2341T, 1:1000), Chk1 (Cell Signaling 2360S, clone 2G1D5, 1:1000), and Actin loading control. **(b)** Similar cellular localization of XLF-WT and XLF-K160D. *XLF-KO* HEK293 cells were stably transfected with EV, 3x-FLAG-XLF-WT, and 3x-FLAG-XLF-K160D, fixed with 4% paraformaldehyde, quenched with 0.1-M glycine and permeabilized with 0.5% triton-X 100 prior to probing with antibodies against FLAG (Sigma F3165, clone M2, 1:500), secondary antibody (Thermofisher/Invitrogen A-11029, 1:250), and DAPI. Scale bar = 50 μm. Shown are representative images from an experiment performed once.

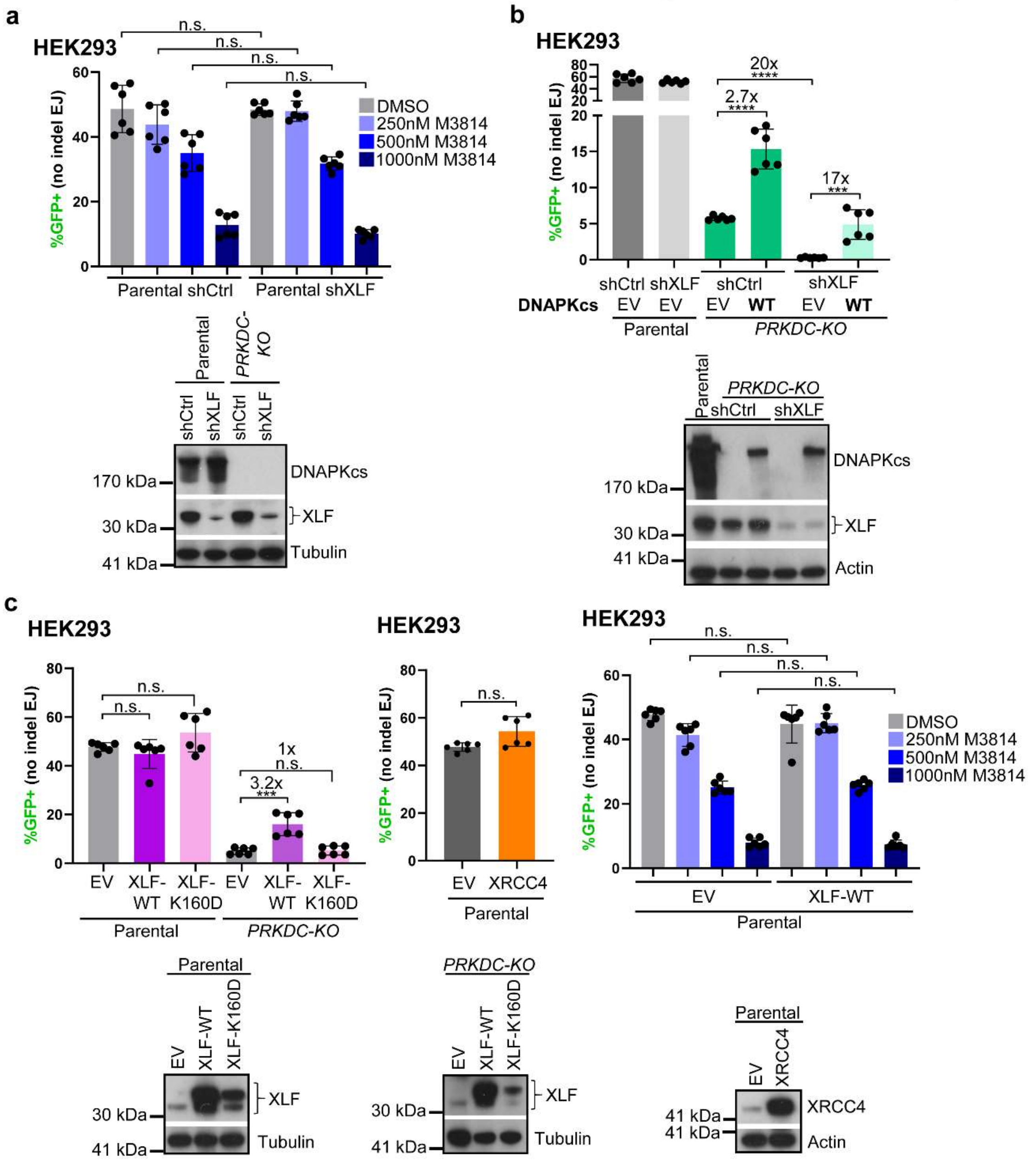

Supplementary Figure 3. Effects of XLF depletion, and overexpression of XLF-WT, XLF-K160D, and XRCC4 on No Indel EJ. (a) Depletion of XLF does not affect the frequency of No Indel EJ, nor the fold-influence of M3814. HEK293 parental and *PRKDC-KO* cell lines were transduced with a lentiviral shRNA vector targeting XLF (shXLF) that causes depletion of XLF as shown in the immunoblots, compared to cells transduced with a non-targeting shRNA vector (shCtrl). Shown is the frequency of No Indel EJ parental cells with shXLF and shCtrl treated with or without M3814. n = 6 biologically independent transfections. Statistics with unpaired two-tailed *t*-test using Holm-Sidak correction. n.s. = not significant. **(b)** Depletion of XLF causes a marked reduction in No Indel EJ in *PRKDC-KO* cells. Shown is the frequency of No Indel EJ in parental and *PRKDC-KO* cells transduced with shCtrl or shXLF, as described in (a), and also transiently transfected with

either DNAPKcs expression vector or EV control. n = 6 biologically independent transfections. Statistics as in (a). ****P<0.0001**,** ***P=0.000264 **(c)** Overexpression of XLF-WT (but not XLF-K160D) causes an increase in No Indel EJ in *PRKDC-KO* cells, but has no such effect on parental cells with or without M3814 treatment, nor does XRCC4 overexpression have an effect on parental cells. Shown are No Indel EJ frequencies for such overexpression experiments. Immunoblots show levels of XLF-WT, XLF-K160D, and XRCC4. n = 6 biologically independent transfections. Statistics as in (a). ***P=0.000492, n.s. = not significant. Data are represented as mean values +/- SD.

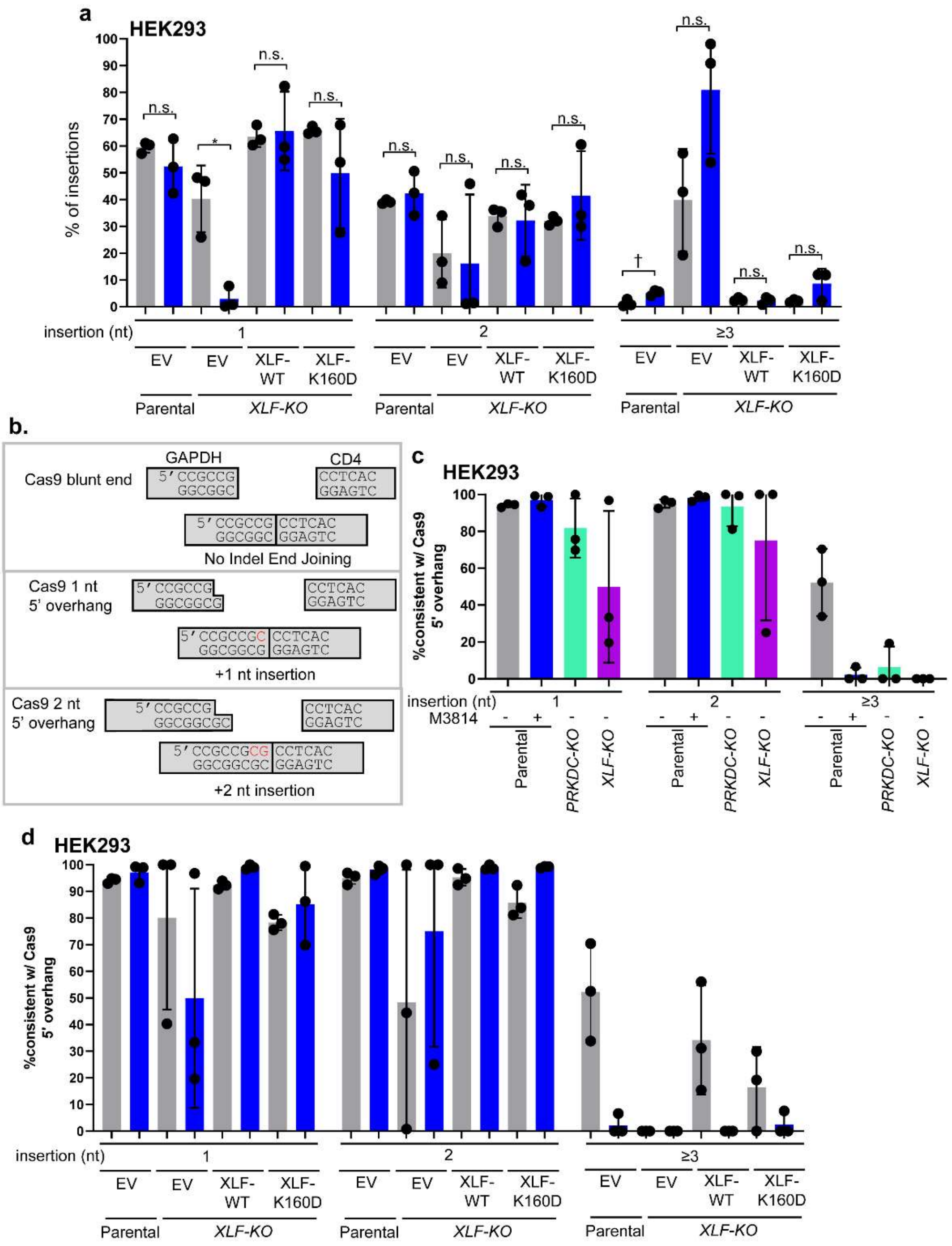

Supplementary Figure 4. GAPDH-CD4 insertions are consistent with Cas9 staggered DSBs causing 5' overhangs. (a) Shown is the frequency of insertion sizes for parental and *XLF-KO* cells treated with or without M3814 for the insertion events shown in Figure 3e. n = 3 biologically independent transfections. Statistics with unpaired two-tailed *t*-test using Holm-Sidak correction. **P*=0.0311, †*P*=0.0151 not significant after correction (unadjusted P-value), n.s. = not significant. **(b)** Shown are predicted blunt EJ outcomes for the GAPDH-CD4 rearrangement following paired blunt DSBs (No Indel EJ), a staggered 1 nt 5' overhang DSB at GAPDH that is filled in (+1 C nucleotide insertion), and a staggered 2 nt overhang DSB at GAPDH that is filled (+2 CG nucleotide insertion). Staggered cleavage

at the *CD4* gene would not be expected to cause GAPDH-CD4 insertion mutations, because the staggered DSB would remove nucleotides on the 3' strand of the distal end, which when filled in, restore the predicted blunt DSB. **(c)** For the insertions shown in Figure 4C (1, 2, ≥3 nts), shown are the frequencies of insertions with a sequence consistent with Cas9 staggered DSBs causing 5' overhangs that is followed by fill-in synthesis and blunt EJ (i.e., the % consistent w/ Cas9 5' overhang). n = 3 biologically independent transfections. **(d)** For the insertions shown in (a), shown is the % consistent w/ Cas9 5' overhang. n = 3 biologically independent transfections. Data are represented as mean values +/- SD.

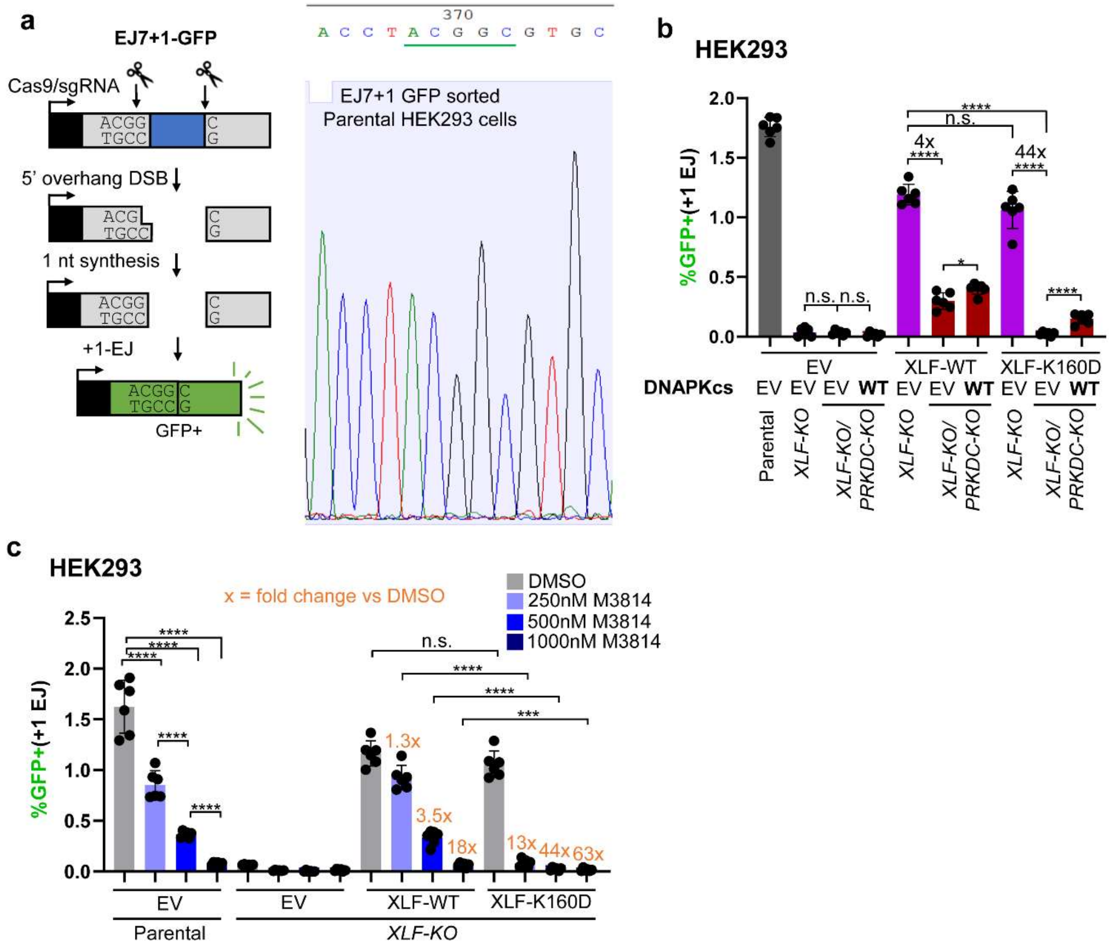

Supplementary Figure 5. EJ with insertions that are likely caused by staggered Cas9 DSBs show similar genetic requirements as No Indel EJ. (a) Validation of the EJ7+1-GFP reporter. Shown is a diagram of the EJ7+1-GFP reporter (not to scale) for EJ with 1 nt insertions, and the Sanger sequence trace of GFP+ cells sorted from HEK293 parental cells using this assay. **(b)** DNAPKcs is required for +1 EJ in cells with XLF-K160D. n = 6 biologically independent transfections. Statistics with unpaired two-tailed *t*-test using Holm-Sidak correction. ****P<0.0001, *P=0.0137 **(c)** Inhibiting DNAPKcs kinase activity has a markedly greater effect on +1 EJ when combined with XLF-K160D. n = 6 biologically independent transfections. Statistics as in (b). ****P<0.0001, ***P=0.000925, n.s. = not significant. Data are represented as mean values +/- SD.

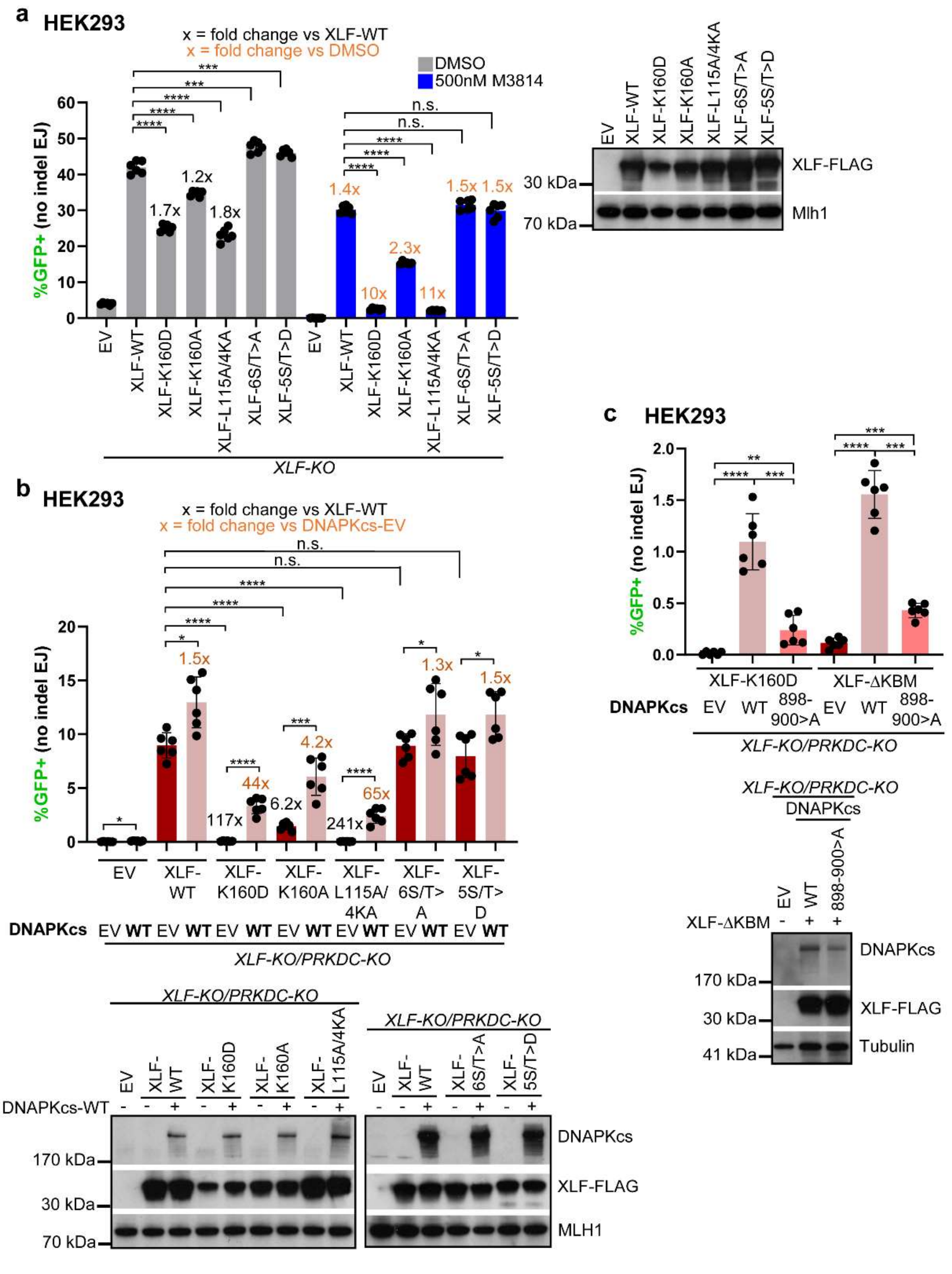

Supplementary Figure 6. DNAPKcs becomes essential to promote No Indel EJ in combination with XLF-K160A and XLF-L115A/4KA) but not mutants with disruption of C-terminal phosphorylation sites (6S/T>A, 5S/T>D), and residues in DNAPKcs implicated in dimerization (898-900) are important for No Indel EJ with a weakened XLF. (a) Shown are effects of 500 nM M3814 treatment on No Indel EJ on four additional mutants of XLF (K160A, L115A/4KA, 6S/T>A, 5S/T>D), with XLF-WT and K160D included for comparison. n = 6 biologically independent transfections. Statistics with unpaired two-tailed *t*-test using Holm-Sidak correction. *****P*<0.0001, XLF-WT

DMSO vs. XLF-6S/T>A DMSO ***P = 0.000251, XLF-WT DMSO vs. XLF-5S/T>D DMSO ***P = 0.00046, n.s. = not significant. Immunoblots shows levels of FLAG-tagged XLF-WT and mutants. MLH1 (abcam ab92312) is used as a loading control. **(b)** Shown are effects of DNAPKcs loss on No Indel EJ in combination with XLF-WT and the mutants shown in (a). Statistics as in (a). ****P<0.0001, ***P=0.000388, XLF-EV EV vs DNAPKcs-WT *P=0.0274, XLF-WT EV vs. DNAPKcs-WT *P=0.0158, XLF-6S/T>A EV vs DNAPKcs-WT *P=0.0439, XLF-5S/T>D EV vs DNAPKcs-WT *P=0.0274, n.s. = not significant. Immunoblots show levels of FLAG-tagged XLF-WT and mutants, and DNAPKcs, with MLH1 as a loading control. n = 6 biologically independent transfections. **(c)** Shown are effects of expressing DNA-PKcs-WT vs. DNAPKcs-898-900>A on No Indel EJ in cells expressing either XLF-K160D, or XLF-ΔKBM. n = 6 biologically independent transfections. Statistics as in (a). Immunoblot shows levels of DNAPKcs-WT, DNAPKcs-898-900>A and XLF-ΔKBM. ****P<0.0001, XLF-ΔKBM EV vs. DNAPKcs-898-900>A ***P=0.000181, XLF-ΔKBM DNAPKcs-WT vs. DNAPKcs-898-900>A ***P=0.000181, XLF-K160D EV vs DNAPKcs-898-900>A **P=0.0035. Error bars = SD. Data are represented as mean values +/- SD.

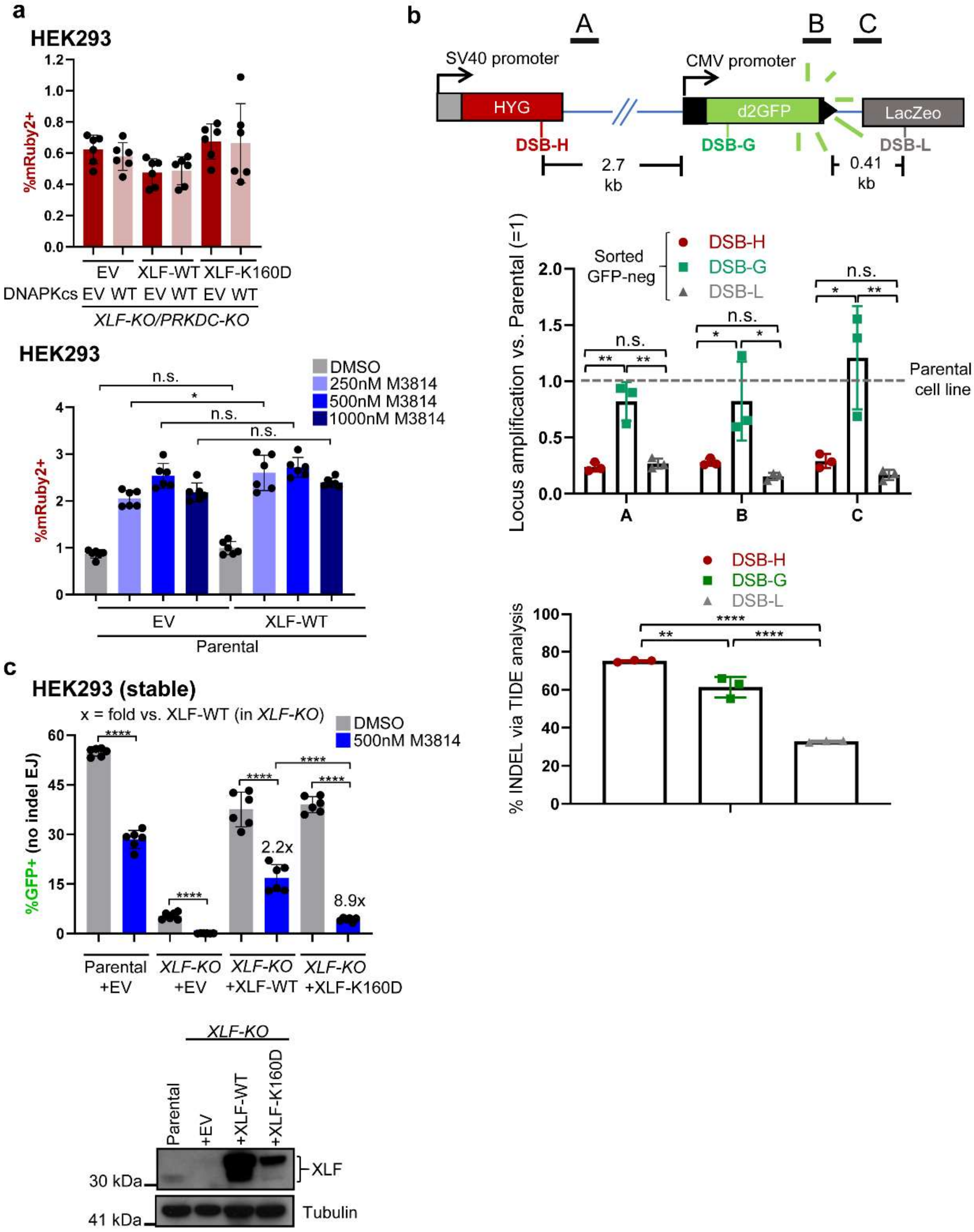

Supplementary Figure 7. HDR analysis in *XLF-KO/PRKDC-KO* and parental cells, validation experiments for the GFPd2-SV reporter, and validation of the XLF-WT and K160D stable HEK293 cell lines. (a) Shown is the frequency of HDR via the LMNA-HDR assay in *XLF-KO/PRKDC-KO* and parental cells with EV or complementation vectors. Also shown is the frequency of HDR in parental cells overexpressing XLF-WT, performed as in Supplementary Fig 3c, which shows the immunoblot validation. n = 6 biologically independent transfections. Statistics with unpaired two-tailed *t*-test using

Holm-Sidak correction. P=0.0364, n.s. = not significant. (**b**) Validation of GFPd2-SV reporter in HEK293 cells. Shown is a diagram of the GFPd2-SV reporter (not to scale) with loci positions examined by qPCR (A, B, and, C, not to scale). GFP-negative (GFP-neg) cells were enriched by cell sorting following DSBs induced by the sgRNAs DSB-H, DSB-G, and DSB-L. Such GFP-neg cells were then examined for amplification of each locus shown (A, B, and C) relative to parental GFP+ cells, using Actin amplification to calculate $2^{-\Delta\Delta Ct}$. n=3 amplifications. Shown is TIDE analysis of samples following DSBs induced by sgRNAs DSB-H, DSB-G, and DSB-L, each using amplification with primers flanking the predicted DSB site. n = 3 biologically independent transfections. One Way Anova with Tukey's post-test. Locus amplification vs. Parental: Site A DSB-H vs. DSB-G **P=0.0012, Site A DSB-G vs. DSB-L **P=0.0017, Site B DSB-H vs. DSB-G *P=0.0393, Site B DSB-G vs. DSB-L *P=0.0164, Site C **P=0.0076, *P=0.0136. n.s.= not significant. %INDEL via TIDE analysis: ****P<0.0001, **P=0.0045. **(c)** Validation of HEK293 stable cell lines using the EJ7-GFP reporter. n = 6 biologically independent transfections. Immunoblots show levels of XLF-WT and XLF-K160D for these stable cell lines. Statistics as in (a) ****P<0.0001. Data are represented as mean values +/- SD.

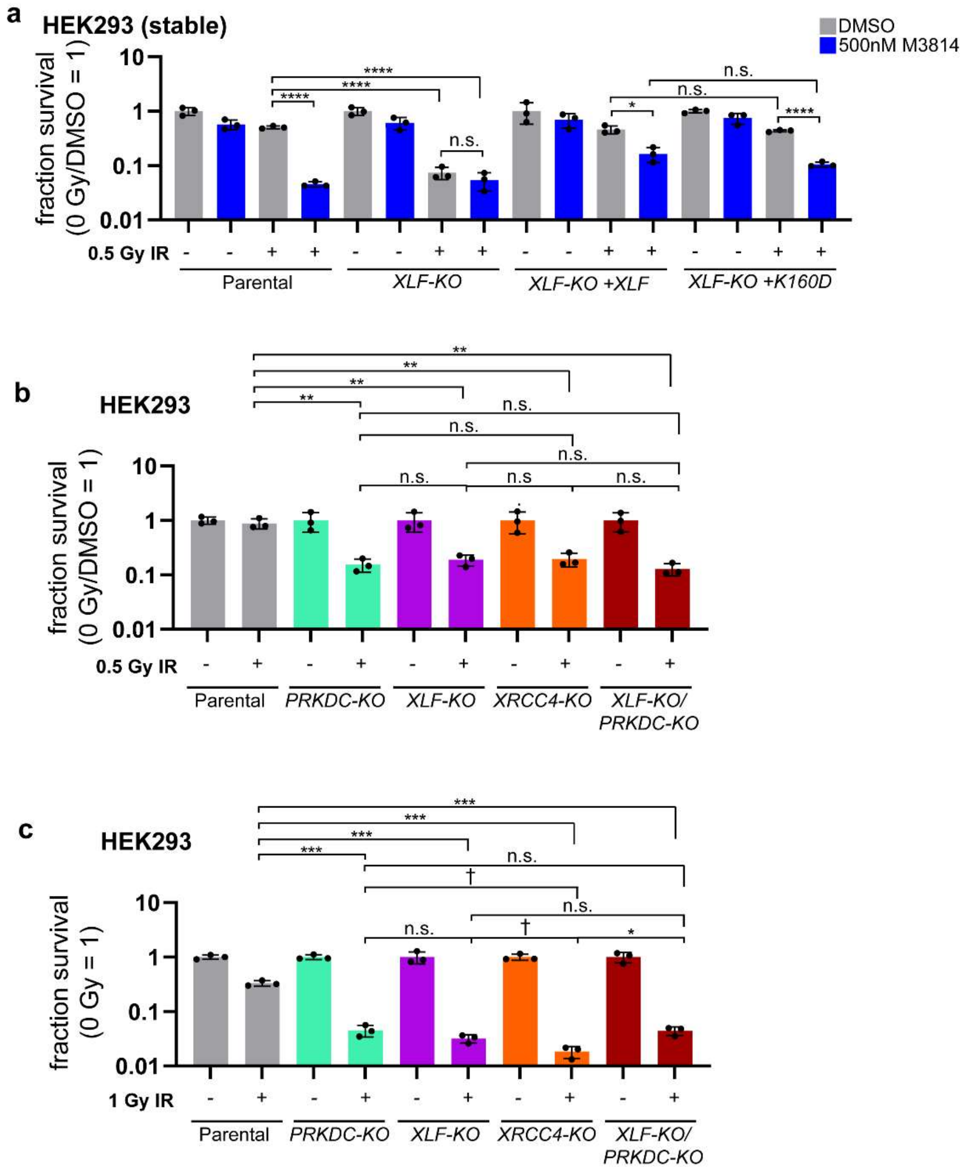

Supplementary Figure 8. IR-sensitivity analysis. a) HEK293 cell lines were treated as in Figure 6f, but using a lower IR dose (0.5 Gy). Namely, cells were treated with DMSO or M3814, and 0 Gy or 0.5 Gy IR dose, and plated to form colonies. Fraction clonogenic survival was determined relative to DMSO 0 Gy for each cell line. n = 3 biologically independent wells of seeded cells. Statistics with unpaired two-tailed *t*-test using Holm-Sidak correction. *****P*<0.0001, **P*=0.0154 n.s.= not significant. **b)** HEK293 cell lines were treated with 0 Gy or 0.5 Gy, and fraction clonogenic survival was determined relative to 0 Gy for each cell line. Treatments included DMSO as in (a) to enable comparison with the M3814 experiments. n = 3 biologically independent wells of seeded cells. Statistics as in (a). Parental DMSO +IR vs. *PRKDC-KO* +IR ***P*=0.00946, Parental DMSO +IR vs. *XLF-KO* +IR ***P*=0.00946, Parental DMSO +IR vs. *XRCC4-KO* +IR ***P*=0.00946, Parental DMSO +IR vs. *XLF-KO/PRKDC-KO* +IR ***P*=0.00946, n.s.= not significant. **c)** Experiment and statistics as in (b) but using 1 Gy treatment. n = 3 biologically independent

wells of seeded cells. Parental DMSO +IR vs. *PRKDC-KO* +IR ****P*=0.000478, Parental DMSO +IR vs. *XLF-KO* +IR ****P*=0.000478, Parental DMSO +IR vs. *XRCC4-KO* +IR ****P*=0.000478, Parental DMSO +IR vs. *XLF-KO*/*PRKDC-KO* +IR ****P*=0.000478, **P*=0.0487, *PRKDC-KO* vs. *XRCC4-KO* †*P*=0.017 not significant after correction (unadjusted *P*-value), *XLF-KO* vs. *XRCC4-KO* †*P*=0.0291 not significant after correction (unadjusted *P*-value), n.s. = not significant. Data are represented as mean values +/- SD.

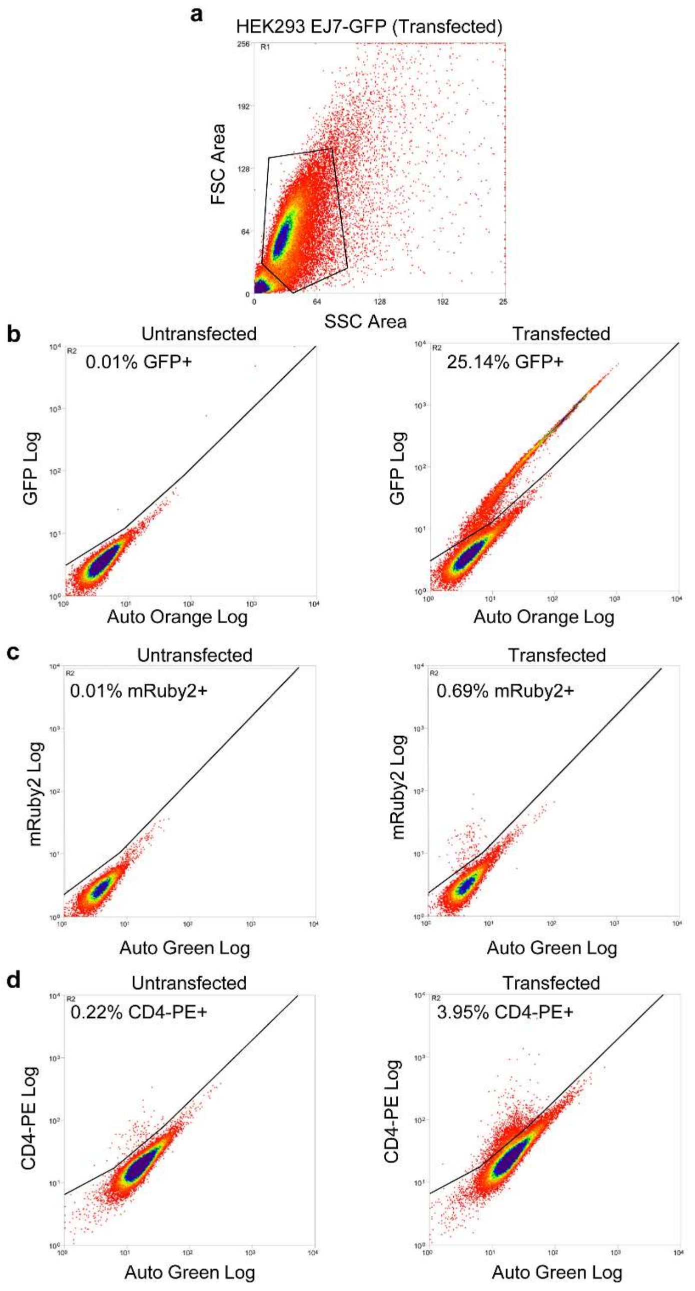

Supplementary Figure 9. Shown are the flow cytometry gating strategies, using the HEK293 EJ7-GFP cell line for the examples. (a) FSC/SSC gating example (shown is the transfected sample in b). FSC/SSC gating is used for analysis of all samples. **(b)** Example of GFP analysis for untransfected control and transfected (7a and 7b Cas9/sgRNA plasmids) cells. **(c)** Example of mRuby2 analysis for untransfected control and transfected (LMNA Cas9/sgRNA and LMNA-mRuby2-Donor plasmids) cells. **(d)** Example of CD4-PE analysis for untransfected control and transfected (GAPDH and CD4 Cas9/sgRNA plasmids) cells. Shown are repair frequencies for these representative plots that demonstrate the gating strategy. As described in the Methods, repair frequencies are normalized to transfection efficiency using parallel wells with the GFP expression vector.
