# Supplementary material for: The importance of DNAPKcs for blunt DNA end joining is magnified when XLF is weakened
Source: Nat Commun. 2022 Jun 27;13:3662. doi: 10.1038/s41467-022-31365-6 (PMC9237100; doi:10.1038/s41467-022-31365-6)
Supplement: Supplementary file 3 — Reporting Summary [file 41467_2022_31365_MOESM3_ESM.pdf]

nature portfolio

Corresponding author(s): Jeremy M Stark, PhD

Last updated by author(s): Jun 1, 2022

Reporting Summary

Nature Portfolio wishes to improve the reproducibility of the work that we publish. This form provides structure for consistency and transparency in reporting. For further information on Nature Portfolio policies, see our Editorial Policies and the Editorial Policy Checklist.

Statistics

For all statistical analyses, confirm that the following items are present in the figure legend, table legend, main text, or Methods section.

n/a	Confirmed	
☐	☒	The exact sample size (n) for each experimental group/condition, given as a discrete number and unit of measurement
☐	☒	A statement on whether measurements were taken from distinct samples or whether the same sample was measured repeatedly
☐	☒	The statistical test(s) used AND whether they are one- or two-sided<br>*Only common tests should be described solely by name; describe more complex techniques in the Methods section.*
☒	☐	A description of all covariates tested
☐	☒	A description of any assumptions or corrections, such as tests of normality and adjustment for multiple comparisons
☐	☒	A full description of the statistical parameters including central tendency (e.g. means) or other basic estimates (e.g. regression coefficient) AND variation (e.g. standard deviation) or associated estimates of uncertainty (e.g. confidence intervals)
☐	☒	For null hypothesis testing, the test statistic (e.g. F, t, r) with confidence intervals, effect sizes, degrees of freedom and P value noted<br>*Give P values as exact values whenever suitable.*
☒	☐	For Bayesian analysis, information on the choice of priors and Markov chain Monte Carlo settings
☒	☐	For hierarchical and complex designs, identification of the appropriate level for tests and full reporting of outcomes
☒	☐	Estimates of effect sizes (e.g. Cohen's d, Pearson's r), indicating how they were calculated

Our web collection on statistics for biologists contains articles on many of the points above.

Software and code

Policy information about availability of computer code

Data collection	Summit 4.4 with the CyAN, and Agilent NovoExpresss Version 1.5.0 with the Quanteon were used to capture flow cytometry data and perform the analysis.
Data analysis	Summit 4.4 with the CyAN, and Agilent NovoExpresss Version 1.5.0 with the Quanteon were used to capture flow cytometry data and perform the analysis. Statistical tests were performed with Prism Version 8.3.0. DNAPKcs-S2056p signals were quantified with ImageJ.

For manuscripts utilizing custom algorithms or software that are central to the research but not yet described in published literature, software must be made available to editors and reviewers. We strongly encourage code deposition in a community repository (e.g. GitHub). See the Nature Portfolio guidelines for submitting code & software for further information.

Data

Policy information about availability of data

All manuscripts must include a data availability statement. This statement should provide the following information, where applicable:

- Accession codes, unique identifiers, or web links for publicly available datasets
- A description of any restrictions on data availability
- For clinical datasets or third party data, please ensure that the statement adheres to our policy

The datasets generated during and/or analyzed during the current study are included in the study and also are available from the corresponding author on reasonable request. The image of the published structure of the XLF homodimer is from publicly available data from Protein Data Bank 2R9A.

Field-specific reporting

Please select the one below that is the best fit for your research. If you are not sure, read the appropriate sections before making your selection.

☒ Life sciences ☐ Behavioural & social sciences ☐ Ecological, evolutionary & environmental sciences

For a reference copy of the document with all sections, see nature.com/documents/nr-reporting-summary-flat.pdf

Life sciences study design

All studies must disclose on these points even when the disclosure is negative.

Sample size	Statistical analysis was not used to predetermine sample size. However, each experiment involved the same sample size for each condition, which was determined at the beginning of the experiment, and all statistical analysis used a sample size of at least three. Indeed, all flow cytometry reporter assay measurements, which is a central approach in the study, each used a sample size of 6.
Data exclusions	No data/experiments were excluded if control transfections were valid (e.g., transfection efficiency).
Replication	This study is based on measurements of DNA repair outcome frequencies, and replicates were performed on all experiments as shown in the figures.
Randomization	These studies did not involve randomization. Covariates for the flow cytometry reporter assay experiments, which is a central approach of this study, were controlled by examining transfection efficiency for each condition in each experiment. Covariates for the clonogenic survival experiments were controlled by examining all cell lines and conditions for a given figure panel in parallel.
Blinding	As described in the Methods, colony counting was performed with sample identity blinded to the experimenter performing the counting. Otherwise, experiments did not involve blinding, because they used automated methods (e.g., flow cytometer software) for the data collection and analysis.

Reporting for specific materials, systems and methods

We require information from authors about some types of materials, experimental systems and methods used in many studies. Here, indicate whether each material, system or method listed is relevant to your study. If you are not sure if a list item applies to your research, read the appropriate section before selecting a response.

Materials & experimental systems

n/a	Involved in the study	
☐	☒	Antibodies
☐	☒	Eukaryotic cell lines
☒	☐	Palaeontology and archaeology
☒	☐	Animals and other organisms
☒	☐	Human research participants
☒	☐	Clinical data
☒	☐	Dual use research of concern

Methods

n/a	Involved in the study	
☒	☐	ChIP-seq
☐	☒	Flow cytometry
☒	☐	MRI-based neuroimaging

Antibodies

Antibodies used	All catalog numbers, clone names (when available), and dilutions for antibodies are provided in the Methods section "Immunoblotting" and/or the supplemental figure legends. We repeat this information here: phycoerythrin-CD4 antibody (BioLegend, 317410, clone OKT4, 1:500), DNAPKcs (Invitrogen MA5-13238, clone 18-2, 1:1000), DNAPKcs-S2056p (Abcam ab124918, clone EPR5670, 1:1000), XLF (Bethyl A300-730A, 1:1000), XRCC4 (Santa Cruz sc271087, clone C-4, 1:1000), Tubulin (Sigma T9026, clone DM1A, 1:1000), FLAG-HRP (Sigma A8592, clone M2, 1:1000), ACTIN (Sigma A2066), HRP goat anti-mouse (Abcam ab205719, 1:3000), and HRP goat anti-rabbit (Abcam ab205718, 1:3000), DNAPKcs, S6-S235/236p (Cell Signaling 2211S, 1:1000), S6 (Cell Signaling 2217S, clone 5G10, 1:1000), ATM-S1981p (Abcam ab81292, clone EP1890Y, 1:1000), ATM (Santa Cruz sc23921, clone 2C1, 1:1000), Chk1-S345p (Cell Signaling 2341T, 1:1000), Chk1 (Cell Signaling 2360S, clone 2G1D5, 1:1000), FLAG (Sigma F3165, clone M2, 1:500), secondary antibody (Thermofisher/Invitrogen A-11029, 1:250).
Validation	Primary antibodies were used in the study to confirm expression/knockout of various gene products. As these are all commercial antibodies, manufacturers' validation statements can be found using the catalog numbers listed, which we also provide here, as follows: phycoerythrin-CD4 antibody (BioLegend, 317410) Reactivity: Human, Cross-Reactivity: Chimpanzee, Cynomolgus, Rhesus. DNAPKcs (Invitrogen MA5-13238) Species Reactivity: Human, Rat. DNAPKcs-S2056p (Abcam ab124918) Reacts with: Human. Does not react with: Mouse, Rat. XLF (Bethyl A300-730A) Reactivity: Human. XRCC4 (Santa Cruz sc271087) detection of XRCC4 of mouse, rat and human origin. Tubulin (Sigma T9026) species reactivity: bovine, rat, yeast, human, mouse, chicken, fungi, amphibian. FLAG-HRP (Sigma A8592) species reactivity: all. ACTIN (Sigma A2066) species reactivity: wide range, human, chicken, amoeba, slime mold, vertebrates. HRP goat anti-mouse (Abcam ab205719) target species: mouse. HRP goat anti-rabbit (Abcam ab205718) target species:

rabbit. S6-S235/236p (Cell Signaling 2211S) Reactivity: Human, Mouse, Rat, Monkey, S. Cerevisiae. S6 (Cell Signaling 2217S) Species Reactivity: Human, Mouse, Rat, Monkey. ATM-S1981p (Abcam ab81292) Reacts with: Human. ATM (Santa Cruz sc23921) Reactivity: Human, Mouse, Rat. Chk1-S345p (Cell Signaling 2341T) Species Reactivity: Human, Mouse, Rat, Monkey. Chk1 (Cell Signaling 2360S) Species Reactivity: Human, Mouse, Rat, Monkey. FLAG (Sigma F3165) Reactivity: all. Secondary antibody (Thermofisher/Invitrogen A-11029) target: mouse.

Eukaryotic cell lines

Policy information about cell lines

Cell line source(s)	All sources of the cell lines are described in the Methods section "Plasmids and Cell Lines," which we repeat here. Several cell lines were described previously: HEK293 Flp-In T-REx cell lines (EJ7-GFP parental line used to generate XLF-KO and XRCC4-KO), U2OS (EJ7-GFP parental and EJ7-GFP XLF-KO), and mESC EJ7-GFP reporter cell lines (WT, Xlf-/-, and Xrcc4-/-)29,31. The vendor of the parental HEK293 Flp-In T-REx cell line is Invitrogen/Thermofisher, which according to their documentation were derived from The American Type Culture Collection number CRL-1573, which are HEK293, not 293T. The Prkdc-/- mESC line was generously provided by Dr. Frederick Alt (Harvard)81. References: (29) Bhargava, R., Lopezcolorado, F. W., Tsai, L. J. & Stark, J. M. The canonical non-homologous end joining factor XLF promotes chromosomal deletion rearrangements in human cells. The Journal of biological chemistry 295, 125-137, doi:10.1074/jbc.RA119.010421 (2020). (31) Bhargava, R. et al. C-NHEJ without indels is robust and requires synergistic function of distinct XLF domains. Nature communications 9, 1-14 (2018). (81) Gao, Y. et al. A Targeted DNA-PKcs-Null Mutation Reveals DNA-PK-Independent Functions for KU in V(D)J Recombination. Immunity 9, 367-376 (1998).
Authentication	All human cells lines (U2OS and HEK293 Flp-In T-REx) were validated by short tandem repeat profiling, as described in prior publications with these cell lines. Specifically in these two publications: Bhargava, R., Lopezcolorado, F. W., Tsai, L. J. & Stark, J. M. The canonical non-homologous end joining factor XLF promotes chromosomal deletion rearrangements in human cells. The Journal of biological chemistry 295, 125-137, doi:10.1074/jbc.RA119.010421 (2020). 31. Bhargava, R. et al. C-NHEJ without indels is robust and requires synergistic function of distinct XLF domains. Nature communications 9, 1-14 (2018).
Mycoplasma contamination	As noted in the Methods section, "cell lines tested negative for mycoplasma contamination."
Commonly misidentified lines (See ICLAC register)	None.

Flow Cytometry

Plots

Confirm that:

☒ The axis labels state the marker and fluorochrome used (e.g. CD4-FITC).

☒ The axis scales are clearly visible. Include numbers along axes only for bottom left plot of group (a 'group' is an analysis of identical markers).

☒ All plots are contour plots with outliers or pseudocolor plots.

☒ A numerical value for number of cells or percentage (with statistics) is provided.

Methodology

Sample preparation	Cells were trypsinized and fixed with formaldehyde at a final concentration of 3%. For CD4 analysis, the cells were first stained with CD4-PE antibody (BioLegend, 317410)), as described in the Methods.
Instrument	As described in the Methods, the Dako CyAN ADP, or ACEA Quanteon cytometer were used.
Software	Summit 4.4 software was used for the CyAN, and Agilent NovoExpresss Version 1.5.0 for the Quanteon.
Cell population abundance	N/A for these experiments that involve determining frequency of events.
Gating strategy	Cells were gated using FSC/SSC, excluding only high/low events in either parameter, and was set as consistent for each experiment. These gated cells were then evaluated for GFP+/-, mRuby2+/-, or CD4-PE+/-, each with the gating strategies shown in Supplemental Figure S9.

☒ Tick this box to confirm that a figure exemplifying the gating strategy is provided in the Supplementary Information.
